# Nanobiotechnology applications of reconstituted high density lipoprotein

**DOI:** 10.1186/1477-3155-8-28

**Published:** 2010-12-01

**Authors:** Robert O Ryan

**Affiliations:** 1 Center for Prevention of Obesity, Cardiovascular Disease and Diabetes, Children's Hospital Oakland Research Institute, 5700 Martin Luther King Jr. Way, Oakland CA 94609, USA; 2 Department of Nutritional Sciences and Toxicology University of California at Berkeley, USA

## Abstract

High-density lipoprotein (HDL) plays a fundamental role in the Reverse Cholesterol Transport pathway. Prior to maturation, nascent HDL exist as disk-shaped phospholipid bilayers whose perimeter is stabilized by amphipathic apolipoproteins. Methods have been developed to generate reconstituted (rHDL) *in vitro *and these particles have been used in a variety of novel ways. To differentiate between physiological HDL particles and non-natural rHDL that have been engineered to possess additional components/functions, the term nanodisk (ND) is used. In this review, various applications of ND technology are described, such as their use as miniature membranes for solubilization and characterization of integral membrane proteins in a native like conformation. In other work, ND harboring hydrophobic biomolecules/drugs have been generated and used as transport/delivery vehicles. In vitro and in vivo studies show that drug loaded ND are stable and possess potent biological activity. A third application of ND is their use as a platform for incorporation of amphiphilic chelators of contrast agents, such as gadolinium, used in magnetic resonance imaging. Thus, it is demonstrated that the basic building block of plasma HDL can be repurposed for alternate functions.

## Background

The term high-density lipoprotein (HDL) describes a continuum of plasma lipoprotein particles that possess a multitude of different proteins and a range of lipid constituents [1]. The major physiological function of HDL is in Reverse Cholesterol Transport [RCT; [2]]. The well-documented inverse relationship between plasma HDL concentration and incidence of cardiovascular disease has generated considerable interest in development of strategies to increase HDL levels. Aside from exercise, moderate consumption of alcohol and a healthy lifestyle, pharmacological approaches are being pursued with the goal of enhancing athero-protection [3]. In addition to these strategies, direct infusion of reconstituted HDL (rHDL) into subjects has been performed [4]. The idea is that parenteral administration of rHDL will promote RCT, facilitating regression of atheroma. Indeed, Nissen et al. [5] reported Phase II clinical trial results showing a decrease in intimal thickness in patients treated with rHDL harboring a variant apolipoprotein A-I.

**Figure 1:**
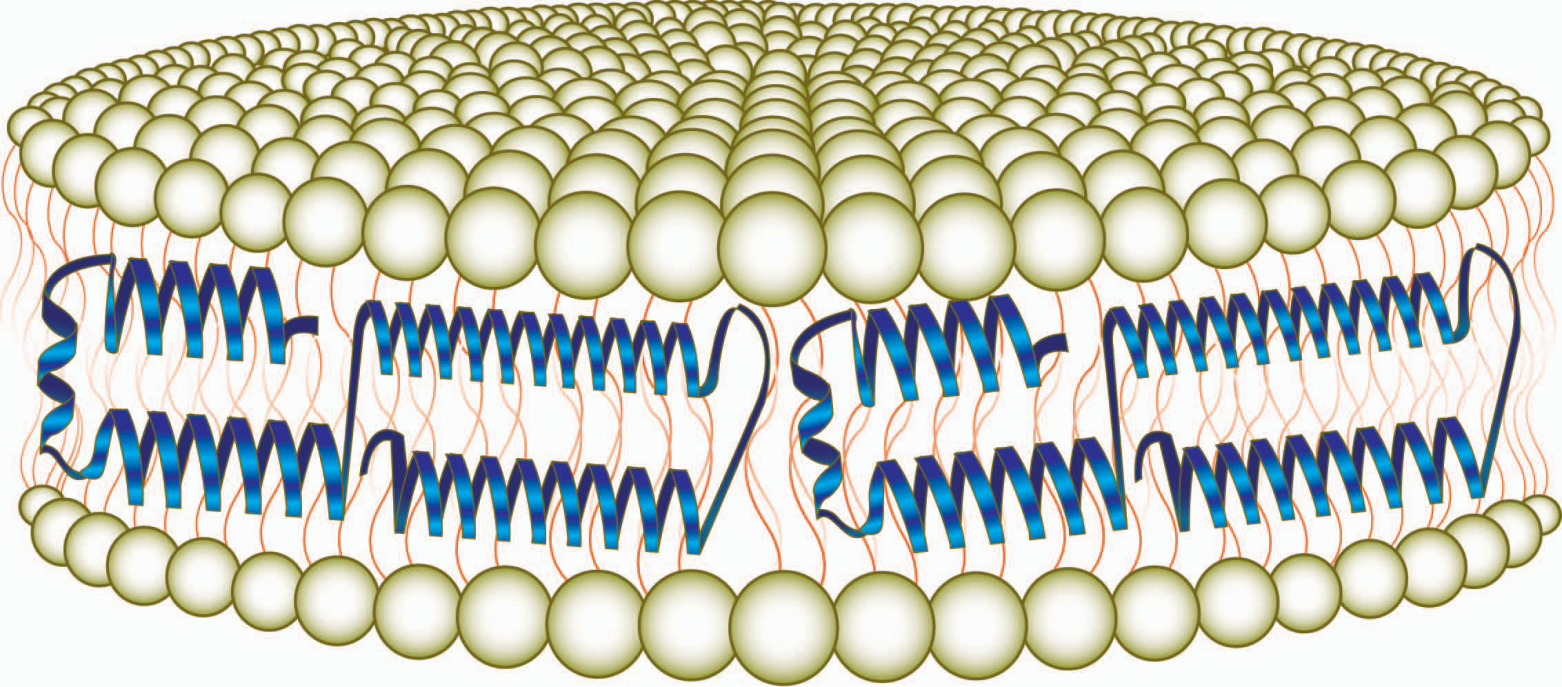
**Schematic diagram of rHDL structural organization**. The complex depicted is comprised of a disk-shaped phospholipid bilayer that is circumscribed by an amphipathic "scaffold" protein. Note: The exact structural organization of rHDL remains controversial. Recently, evidence consistent with an ellipsoidal shape has been presented [59-61].

While its structural properties and composition can be rather complex, in its most basic form, HDL are relatively simple, containing only phospholipid and apolipoprotein (apo). The most abundant and primary apolipoprotein component of plasma HDL is apoA-I. Human apoA-I (243 amino acids) is well characterized in terms of its structural and functional properties. When incubated with certain phospholipid vesicles *in vitro*, apoA-I induces formation of rHDL. The key structural element of apoA-I required for rHDL assembly is amphipathic α-helix. Indeed, other apolipoproteins, apolipoprotein fragments or peptides that possess this secondary structure, can also combine with phospholipid to form rHDL. In general, the product particle is a nanometer scale disk-shaped phospholipid bilayer whose periphery is circumscribed by two or more apolipoprotein molecules (Figure 1). Indeed, a defining characteristic of members of the class of exchangeable apolipoprotein is an ability to form rHDL. For the purpose of this review, the protein/peptide component of discoidal rHDL is termed the "scaffold" in recognition of its function in stabilization of the otherwise unstable edge of the bilayer.

## Production of rHDL

Detailed structure-function studies of exchangeable apolipoproteins have given rise to two general methods for discoidal rHDL formation: detergent dialysis and direct conversion. Whereas the detergent dialysis method [6] has the advantage that a broad spectrum of bilayer forming phospholipids can be employed, a disadvantage relates to the potentially problematic detergent removal step, which can be achieved by specific absorption or exhaustive dialysis. On the other hand, while limited to fewer phospholipid substrates, the direct conversion method does not employ detergents. The types of phospholipids commonly used in the direct conversion method are synthetic, saturated acyl chain glycerophospholipids such as dimyristoylphosphatidylcholine (DMPC) or dimyristoylphosphatidylglycerol. These lipids undergo a gel to liquid crystalline phase transition in the range of 23°C. Normally, the phospholipid substrate is hydrated and induced to form vesicles, either by membrane extrusion or sonication. Incubation of the phospholipid vesicle substrate with an appropriate scaffold protein (e.g. apoA-I) induces self-assembly of rHDL. It is likely that the reaction proceeds most efficiently in this temperature range because defects created in the vesicle bilayer surface serve as sites for apolipoprotein penetration, bilayer disruption and transformation to rHDL. Among the apolipoproteins that have been examined for their ability to transform phospholipid bilayer vesicles into rHDL and function as a scaffold are apoA-I, apoE, apoA-IV, apoA-V and apolipophorin III. In addition, it is known that fragments of apolipoproteins [7] or designer peptides [8] can substitute for full-length apolipoproteins in this reaction. Based on this description, it is evident that myriad combinations of phospholipid and scaffold can be employed to formulate unique rHDL. These particles are readily characterized in terms of size by non-denaturing polyacrylamide gel electrophoresis and morphology by electron or atomic force microscopy (AFM).

Over the past decade, discoidal rHDL have been repurposed for applications beyond its physiological role in lipoprotein metabolism. This review describes active areas of research that have evolved from our basic understanding of rHDL structure and assembly. Whereas rHDL has been modified to re-task it for alternate purposes, its basic structural elements, including disk shape, nanometer scale size and a planar bilayer whose periphery is stabilized by a scaffold, are preserved. In this manner, rHDL serve as a platform capable of packaging transmembrane proteins in a native-like membrane environment, solubilization and delivery of hydrophobic drugs/biomolecules and presentation of contrast agents for magnetic resonance imaging of atherosclerotic lesions. In an effort to distinguish engineered rHDL from classical rHDL, the term nanodisk (ND) is used to describe rHDL formulated to possess a transmembrane protein, drug or non-natural hydrophobic moiety.

## ND as a miniature membrane environment for solubilization of transmembrane proteins

**Figure 2:**
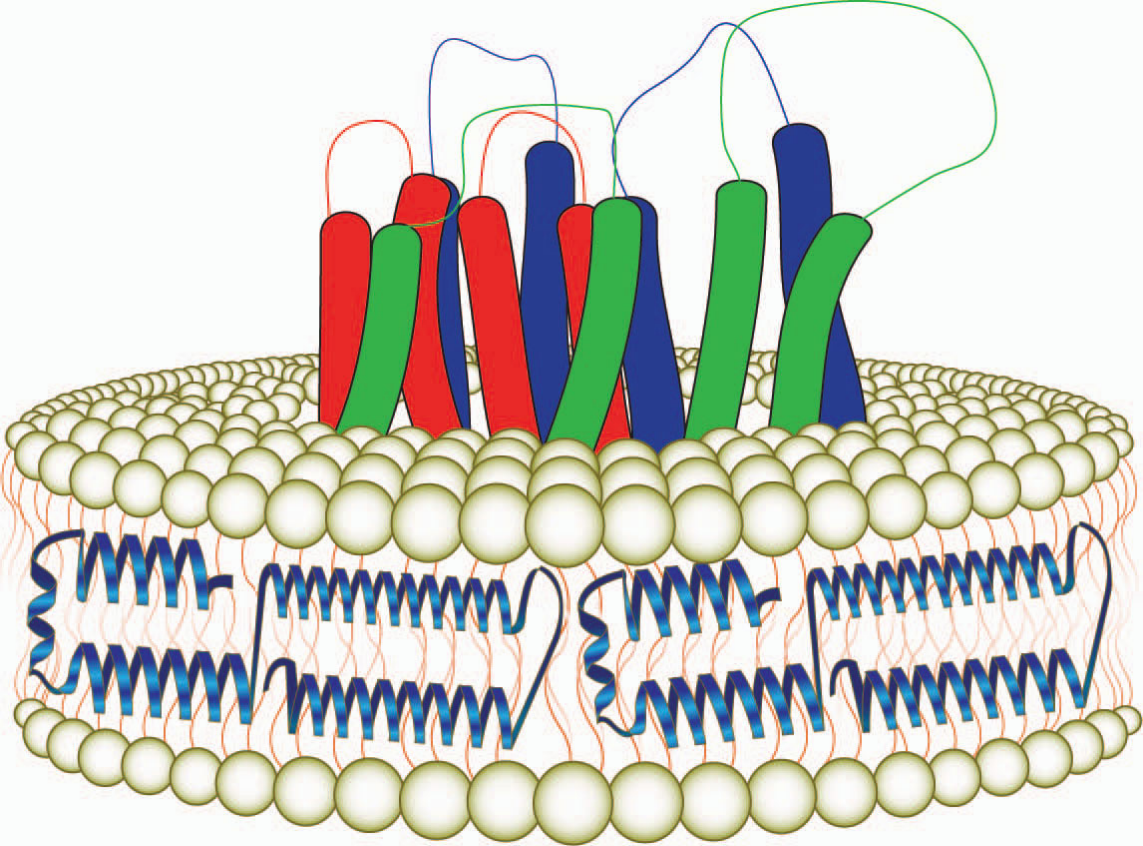
**Diagram of a ND particle with an embedded transmembrane protein**. The bilayer component of the ND provides a miniature bilayer membrane that can accommodate one or more transmembrane proteins in a native-like conformation.

The bilayer component of ND provides a native-like environment for study of transmembrane proteins in isolation. The concepts being developed on this research front are that Type 1, Type 2 or Type 3 membrane proteins can be inserted into ND with retention of their native conformation/biological activity. As with a cell membrane, the inserted protein would align such that its transmembrane segment(s) spans the bilayer while their soluble, extra-membranous portions, exist in the aqueous environment (Figure 2). If correctly inserted, it is anticipated that specific biological or enzymatic properties of the protein will be preserved. The surface area of a 20 nm diameter ND particle is ~300 nm^2^, ample area to accommodate several molecules of a multiple pass transmembrane protein. Advantages of ND versus detergent micelles include a more natural environment and the absence of detergent related effects on conformation or activity of the subject protein. While liposomes are amenable to study of transmembrane proteins, these complexes suffer from having an inaccessible inner aqueous space, protein orientation issues, size variability and lack of complete solubility.

Several groups have successfully generated membrane protein-containing ND, including cytochrome P450 s, seven-transmembrane proteins, bacterial chemoreceptors and others [[9-12] for reviews]. Advantages of ND for this purpose include particle size homogeneity, access to both sides of the membrane and greater control over the oligomerization state of the inserted protein. The power and potential of this technology is illustrated by the following specific examples:

### a. Bacteriorhodopsin

Bacteriorhodopsin (bR) from the purple membrane of *halobacterium halobium *is a prototype integral membrane protein. This 247 amino acid, light-driven proton pump possesses a covalently bound molecule of retinal. Elegant electron crystallography methods were developed and employed by Henderson and Unwin to decipher the structure of bacteriorhodopsin at near atomic resolution [13]. The protein is comprised of a bundle of seven ~25 residue α-helical rods that span the bilayer while charged residues at the surface of the membrane contact the aqueous solvent. In its native form bR exists as trimers that organize into a two-dimensional hexagonal array in the plane of the membrane. In 2006, Bayburt et al. [14] assembled bR into ND. Under the conditions employed each ND contained three bR molecules. Small angle X-ray scattering analysis provided evidence that bR embeds in the ND bilayer while evidence of trimer formation was obtained by near UV circular dichroism spectroscopy of the retinal absorbance bands. In further study of this system, Blanchette et al. [15] used atomic force microscopy to image and analyze bR-ND. The self-assembly process employed by these authors generated two distinct ND populations, bR-ND and empty-ND, as distinguished by an average particle height increment of 1.0 nm for bR-ND. When bR is present during assembly, ND diameters are larger suggesting the inserted protein influences the dimensions of the product ND.

### b. Cytochrome P450

Baas and coworker [16] reported on structural and functional characterization of cytochrome P450 3A4 (CYP 3A4)-ND. Solution small angle X-ray scattering of CYP 3A4-ND provided evidence that CYP 3A4 retains hydroxylation activity. In other work, Das and Sligar [17] incorporated cytochrome P450 reductase (CPR) into ND and investigated its ability to transfer electrons from NADPH to microsomal P450 s. The redox potential of CPR's FMN and FAD cofactors shifted to more positive values in ND compared to a solubilized version of the reductase in which the N-terminal membrane spanning domain was cleaved. Moreover, when anionic lipids were used to alter the membrane composition of CPR-ND, the redox potential of both flavins became more negative, favoring electron transfer from CPR to cytochrome P450.

### c. ß2-adrenergic receptor

Leitz et al. [18] reported on ND harboring ß2-adrenergic receptor. Evidence that the receptor adopts a native like conformation within the ND milieu was obtained from study of its G-protein coupling activity.

### d. Hydrogenase

Baker et al. [19] reported the physical characterization and hydrogen-evolving activity of ND assembled with hydrogenase obtained from the thermophilic Archea, *Pyrococcus furiosus*. Insofar as this class of membrane bound enzyme is capable of *ex vivo *hydrogen production from starch or glucose, this work may impact development of bioengineered hydrogen generation methods for renewable energy production.

### e. SecYEG

In bacteria, protein transit across the cytoplasmic membrane is mediated by translocase [20]. Translocase consists of the transmembrane protein conducting channel, SecYEG, a soluble motor protein, SecA, and the chaperone, SecB. Nascent proteins destined for secretion are bound by SecB and directed to SecYEG- associated SecA. Protein translocation is subsequently driven by SecA through repeated cycles of ATP binding and hydrolysis wherein the target protein is threaded through the SecYEG pore. Alami et al. [21] successfully reconstituted SecYEG into ND and used these particles to study the interaction of SecYEG and its cytosolic partner, SecA. SecYEG-ND were able to trigger dissociation of SecA dimers and associate with the SecA monomer, leading to activation of SecA ATPase. Thus, SecYEG-ND represent a novel means to investigate the role of bacterial protein transport via translocase.

### f. Anthrax toxin

Katayama et al. [22] obtained structural insight into the mechanism whereby protective antigen (PA) pore formation mediates translocation of the enzymatic components of anthrax toxin across membranes. Two populations of PA pores, in vesicles and ND, were reconstructed from electron microscopic images at 22 Å resolution. Fitting the X-ray crystallographic coordinates of the PA pre-pore revealed a prominent flange, formed by convergence of mobile loops that function in protein translocation. Identification of the location of functional elements of the PA pore from electron microscopic characterization of ND embedded PA represents an innovative use of ND technology.

### g. VDAC-1

The voltage-dependent anion channel (VDAC) is an essential protein in the eukaryotic outer mitochondrial membrane, providing a pore for substrate diffusion. High-resolution structures of VDAC-1 in detergent micelles and bicelles have been reported using solution NMR and X-ray crystallography. These studies have resolved longstanding issues related to VDAC membrane topology and provide the first eukaryotic ß-barrel membrane protein structure. At the same time, the structure - function basis for the voltage gating mechanism of VDAC-1 or its modulation by NADH remain unresolved. To address these issues Raschle et al. [23] conducted electron microscopy and solution NMR spectroscopy on VDAC-1-ND. Electron microscopy provided evidence for formation of VDAC-1 multimers, while high-resolution NMR spectroscopy revealed that VDAC-1 is properly folded and manifests NADH binding activity. Thus, ND offer a new approach for study of the biophysical properties of VDAC-1 under native-like conditions.

### h. Hemagglutinin

Influenza virus infection causes significant mortality and morbidity in human populations. Hemagglutinin (HA) is the major protein target of the protective antibody response induced by influenza viral infection. The influenza virion grows by budding from the plasma membrane of an infected cell. The outer envelope of influenza virus consists of a lipid bilayer into which the integral membrane glycoprotein, HA, inserts. Whereas recombinant HA is relatively easy to produce, its efficacy as a vaccine is limited by an inability to retain a native, membrane-bound conformation. Bhattacharya et al. [24] generated recombinant HA-ND (influenza virus strain A/New Caledonia/20/99; H1N1) and investigated its ability to confer immunity upon influenza virus challenge. HA-ND vaccination induced a robust antibody response with a high hemagglutination inhibition titer. The finding that HA-ND vaccination conferred a level of protection comparable to Fluzone^® ^and FluMist^® ^following H1N1 challenge, suggests this approach is worth pursuing in greater detail.

## Vehicle for solubilization and delivery of hydrophobic biomolecules

**Figure 3:**
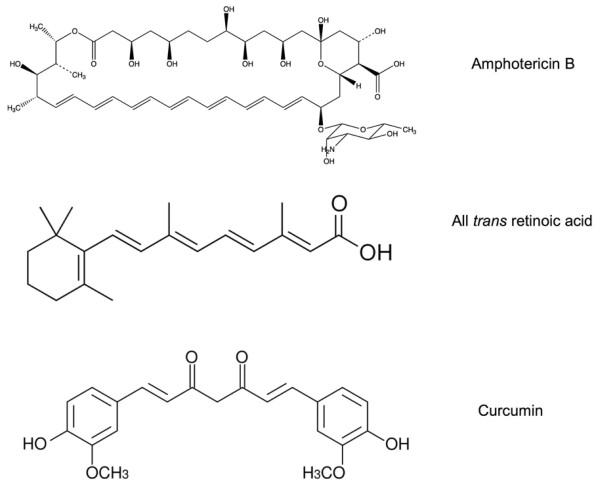
**Structure of small hydrophobic molecules**. The water insoluble molecules shown, including amphotericin B, all trans retinoic acid and curcumin, have been successfully incorporated in ND with retention of biological activity.

Aside from study of membrane bound proteins, another application of ND technology is as a vehicle for transport/delivery of small hydrophobic biomolecules/drugs [25]. To date, several bioactive compounds, including the macrolide polyene antibiotic, amphotericin B (AMB), the isoprenoid, all *trans *retinoic acid (ATRA) and the polyphenol, curcumin, have been successfully integrated into the ND milieu (Figure 3). On the basis of studies characterizing drug incorporation efficiency, retention of biological activity and ease of formulation, it is apparent that ND constitute a platform for solubilization, transport and delivery of hydrophobic bioactive molecules. Recent success in the design and production of targeted-ND offer a means to expand the capability of this approach [26].

### Amphotericin B

AMB has been used clinically for nearly half a century. It is an amphoteric molecule that interacts with membrane sterols (preferably 24 substituted sterols such as ergosterol), forming pores that facilitate leakage of cell contents. Clinical application of this potent antifungal is limited by poor oral bioavailablility, infusion-related toxicity and nephrotoxicity [27]. Using the direct solubilization method, AMB-ND have been formulated with high incorporation efficiency [28,29]. AMB-ND inhibited growth of *Saccharomyces cerevisiae *as well as several pathogenic fungal species [28]. Furthermore, compared to AMB-deoxycholate, AMB-ND display attenuated red blood cell hemolytic activity and decreased toxicity toward cultured hepatoma cells. *In vivo *studies in immunocompetent mice revealed that AMB-ND are nontoxic at concentrations up to 10 mg/kg AMB, and show efficacy in a mouse model of candidiasis at concentrations as low as 0.25 mg/kg [28]. Taken together, these results indicate that AMB-ND constitute a novel formulation that effectively solubilizes the antibiotic and elicits strong *in vitro *and *in vivo *antifungal activity, with no observed toxicity at therapeutic doses.

AMB-ND have also been examined for efficacy in *Leishmania major *infected mice [30,31]. Membranes of these protozoal parasites contain episterol and, as such, are susceptible to AMB. When *L. major*-infected mice were treated with AMB-ND, enhanced efficacy was observed. Mice administered AMB-ND at 1 or 5 mg/kg displayed decreased lesion size and parasite burden. At 5 mg/kg AMB-ND induced complete clearance of the infection, with no lesions remaining and no parasites isolated from infected animals. By contrast, liposomal AMB, at the same dose, was far less effective. The ability of AMB-ND to induce clearance of *L. major *parasites from a susceptible strain of mice without an appreciable change in cytokine response suggests AMB-ND represent a potentially useful formulation for treatment of intrahistiocytic organisms.

### All trans retinoic acid

Retinoids, such as ATRA, are useful agents in cancer therapy as they exhibit a central role in cell growth, differentiation, and apoptosis [32,33]. Its beneficial actions have been well documented for treatment of acute promyelocytic leukemia [34]. ATRA binding to nuclear hormone receptors transactivates target genes, leading to cell growth arrest or apoptosis [35-37]. At the same time, ATRA is insoluble in water, toxic at higher doses and has limited bioavailability [38]. Pharmacological levels can cause retinoic acid syndrome and neurotoxicity, particularly in children [39]. Redmond et al. [40] formulated ATRA into ND. Subsequently, Singh et al. [41] evaluated effects of ATRA-ND on Mantle cell lymphoma (MCL), a subtype of non-Hodgkin's lymphoma that arises from uncontrolled proliferation of a subset of pregerminal center cells located in the mantle region of secondary follicles [42]. In cell culture studies, compared to free ATRA, ATRA-ND more effectively induced reactive oxygen species generation and led to a greater degree of cell death. Mechanistic studies revealed that ATRA-ND enhanced G1 growth arrest, up-regulated p21and p27 and down-regulated cyclin D1. At ATRA concentrations that induce apoptosis, expression levels of retinoic acid receptor-α and retinoid X receptor-γ increased. Taken together, evidence indicates that incorporation of ATRA into ND enhances the biological activity of this retinoid.

### Curcumin

Known chemically as diferuloylmethane, curcumin is a hydrophobic polyphenol derived from rhizome of turmeric (*Curcuma longa*), an East Indian plant. Curcumin possesses diverse pharmacologic effects including anti-inflammatory, anti-oxidant and anti-proliferative activities [43,44]. Furthermore, curcumin is non-toxic, even at relatively high doses [45]. Despite this, clinical advancement of curcumin has been hindered by poor water solubility, short biological half-life and low bioavailability following oral administration. Ghosh et al. [46] formulated curcumin-ND at a 6:1 phospholipid:curcumin molar ratio. When formulated in ND, curcumin is water-soluble and gives rise to a characteristic absorbance spectrum. AFM analysis revealed curcumin-ND are disk-shaped particles with a diameter < 50 nm. In cell culture studies, curcumin-ND induced enhanced HepG2 cell growth inhibition compared to free curcumin. Moreover, curcumin-ND were a more potent inducer of apoptosis in cultured MCL cells than free curcumin.

## Contrast agent enriched ND for medical imaging

**Figure 4:**
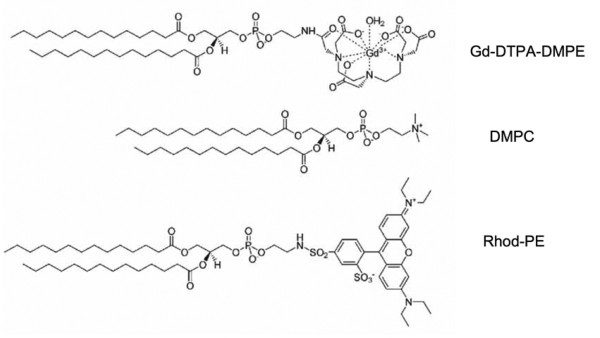
**Structures of specialized lipids**. Gd^3+^-DTPA-DMPE (diethylenetriaminepentaacetate-dimyristoylphosphatidylethanolamine) is an amphiphilic Gd^3+ ^chelator useful in magnetic resonance imaging; DMPC (dimyristoylphosphatidylcholine) is a glycerophospholipid commonly employed as a structural lipid in ND; Rhod-PE (1,2-dimyristoyl-*sn*-glycero-3-phosphoethanolamine-*N*-(lissamine rhodamine B sulfonyl)ammonium salt) is a lipophilic fluorophore useful in fluorescence imaging techniques.

Given that cardiovascular disease is the major cause of mortality in North America, there is a pressing need for noninvasive imaging of atherosclerotic lesions. One of the most promising techniques currently available is magnetic resonance imaging (MRI). In the case of cardiovascular disease, MRI can be used to identify and characterize plaque deposits. In this way it facilitates diagnosis, choice of therapy as well as assessment of the effectiveness of a given intervention. The utility of MRI is significantly enhanced by the use of paramagnetic ions [47]. A popular paramagnetic ion used as a contrast agent for MRI is the chemical element gadolinium (Gd; atomic number 64). Gd^3+ ^chelates are widely used because they provide positive contrast (imaging brightening) in anatomical images rather than negative contrast. Furthermore, Gd has no known biological role and Gd^3+^-chelates are generally considered nontoxic. An example of an amphiphilic Gd^3+ ^chelator is diethylenetriaminepentaacetate-dimyristoylphosphatidylethanolamine (Gd^3+^-DTPA-DMPE) (Figure 4). The lipophilic DMPE moiety of this chelator provides a means to tether Gd^3+ ^to ND. In addition to amphiphilic Gd^3+ ^chelates, ND have also been modified with lipophilic fluorophores, extending their use to fluorescence imaging techniques.

Skajaa et al. [48] have summarized progress toward establishing ND as a vehicle for delivery of diagnostic agents to vulnerable atherosclerotic plaques in mouse models of atherosclerosis. For example, Frias et al. [49] injected Gd^3+^-ND into mice with atherosclerotic lesions. Subsequent MRI analysis revealed a clear enhancement of plaque contrast. Likewise, Cormode et al. [50] used Gd^3+^-ND to enhance contrast in macrophage-rich areas of plaque in a mouse model of atherosclerosis. Cormode et al. [51] incorporated gold, iron oxide, or quantum dot nanocrystals into ND for computed tomography, magnetic resonance, and fluorescence imaging, respectively. By including additional probes in these particles, unique functionalities were introduced. Importantly, the *in vitro *and *in vivo *behavior of such ND mimicked the behavior of native HDL.

Chen et al. [52] introduced a targeting moiety into Gd^3+^-ND in an effort to improve macrophage uptake. A carboxyfluorescein-labeled apoE-derived peptide, termed P2fA2, was used as scaffold in Gd^3+^-ND. Macrophage uptake was studied in J774A.1 macrophages and MRI studies were performed in apoE (-/-) mice. *In vivo *studies showed a more pronounced and significantly higher signal enhancement with the apoE peptide while confocal microscopy studies revealed that P2fA2 Gd^3+^-ND co-localize with intraplaque macrophages. In another application, Chen et al. [53] functionalized Gd^3+^-ND with an α,ß 3-integrin-specific pentapeptide as a means to target ND to angiogenic endothelial cells. Subsequent studies revealed preferential uptake of the targeted ND by endothelial cells.

## Other applications

**Figure 5:**
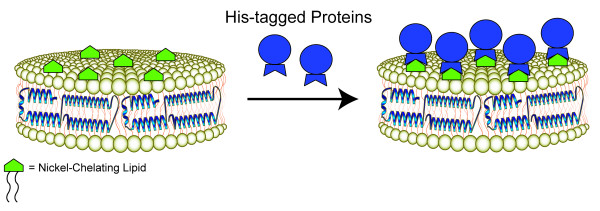
**Capturing His-tagged proteins on the surface of ND**. Incorporation of the nickel-chelating lipid, DOGS-NTA-Ni (1,2-dioleoyl-sn-glycero-3-{[N-(5-amino-1-carboxypentyl) iminodiacetic acid] succinyl}(nickel salt) into ND confers the ability to stably and specifically bind His tagged proteins.

As the applications described above continue to be developed and improved, additional new uses of ND technology have emerged recently. For example, Fischer et al. [54] incorporated synthetic nickel-chelating lipids into ND and examined their ability to bind His-tagged proteins (Figure 5). The nickel-chelating lipid, DOGS-NTA-Ni (1,2-dioleoyl-sn-glycero-3-{[N-(5-amino-1-carboxypentyl) iminodiacetic acid] succinyl}(nickel salt), was incorporated into ND at varying amounts. Gel filtration chromatography, native PAGE and AFM analysis revealed that His-tagged proteins bind to these modified ND in a nickel-dependent manner. In an example of the utility of this approach, DOGS-NTA-Ni-ND were employed as a substrate for binding His-tagged West Nile virus envelope protein [55]. The observation that envelope protein immunogenicity increased upon conjugation to ND suggests they may be useful as a vaccine to prevent West Nile encephalitis. In a modification of this general approach Borch et al. [56] generated ND harboring ganglioside GM_1_. Subsequent studies with GM_1_-ND showed they possess the capacity to recognize and bind its soluble interaction partner, cholera toxin B subunit. Finally, sphingosine-1-phosphate (S1P) is a naturally occurring bioactive lipid that elicits effects on mitogenesis, endothelial cell motility, cell survival and differentiation. Matsuo et al. [57] examined the effect of S1P-ND on tube formation in endothelial cells. The effect of S1P-ND on endothelial cells observed in this study vividly illustrates the utility of incorporating bioactive lipids into the ND platform.

## Conclusions and Future Directions

Emerging from basic studies of HDL metabolism is new technology built around the basic structure of nascent HDL particles. A variety of applications, ranging from membrane protein insertion, drug delivery to functionalized lipid incorporation, have led to significant new advances. The utility of ND technology is intimately linked to the ease with which these particles are generated, the water solubility and nanoscale size of the product particles, together with the imagination of the investigator. An example of the latter is the synthesis of bio-mimetic nanoparticles wherein a gold core serves as a template for assembly of a mixed phospholipid bilayer and association of apoA-I [58]. As the examples described in this review document, the future is very bright for ND technology.

## Abbreviations

HDL: high density lipoprotein; rHDL: reconstituted HDL; RCT: reverse cholesterol transport; Apo: apolipoprotein; DMPC: dimyristoylphosphatidylcholine; AFM: atomic force microscopy; ATRA: all *trans *retinoic acid; AMB: amphotericin B.

## Competing interests

The author is a Founder of Lypro Biosciences Inc. and co-author of US Patent application No. 10/778,640 "Lipophilic drug delivery vehicle and methods of use thereof".

## References

[B1] Fielding CJ High Density Lipoproteins 2007 Weinheim: Wiley-VCH

[B2] Rothblat GH Phillips MC High-density lipoprotein heterogeneity and function in reverse cholesterol transport Curr Opin Lipidol 2010 21 229 238 10.1097/MOL.0b013e328338472d 20480549 PMC3215082

[B3] Natarajan P Ray KK Cannon CP High-density lipoprotein and coronary heart disease: current and future therapies J Am Coll Cardiol 2010 55 1283 1299 10.1016/j.jacc.2010.01.008 20338488

[B4] Dudley-Brown S A shot of good cholesterol: synthetic HDL, a new intervention for atherosclerosis J Cardiovasc Nurs 2004 19 421 424 15529065 10.1097/00005082-200411000-00015

[B5] Nissen SE Tsunoda T Tuzcu EM Schoenhagen P Cooper CJ Yasin M Eaton GM Lauer MA Sheldon WS Grines CL Halpern S Crowe T Blankenship JC Kerensky R Effect of recombinant ApoA-I Milano on coronary atherosclerosis in patients with acute coronary syndromes: a randomized controlled trial JAMA 2003 290 2292 2300 10.1001/jama.290.17.2292 14600188

[B6] Jonas A Reconstitution of high-density lipoproteins Methods Enzymol 1986 128 553 582 full_text 3724523 10.1016/0076-6879(86)28092-1

[B7] Weers PM Narayanaswami V Ryan RO Modulation of the lipid binding properties of the N-terminal domain of human apolipoprotein E3 Eur J Biochem 2001 268 3728 3735 10.1046/j.1432-1327.2001.02282.x 11432739

[B8] Datta G Chaddha M Hama S Navab M Fogelman AM Garber DW Mishra VK Epand RM Epand RF Lund-Katz S Phillips MC Segrest JP Anantharamaiah GM Effects of increasing hydrophobicity on the physical-chemical and biological properties of a class A amphipathic helical peptide J Lipid Res 2001 42 1096 1104 11441137

[B9] Nath A Atkins WM Sligar SG Applications of phospholipid bilayer nanodiscs in the study of membranes and membrane proteins Biochemistry 2007 46 2059 2069 10.1021/bi602371n 17263563

[B10] Borch J Hamann T The nanodisc: a novel tool for membrane protein studies Biol Chem 2009 390 805 814 10.1515/BC.2009.091 19453280

[B11] Ritchie TK Grinkova YV Bayburt TH Denisov IG Zolnerciks JK Atkins WM Sligar SG Reconstitution of membrane proteins in phospholipid bilayer nanodiscs Methods Enzymol 2009 464 211 231 full_text 19903557 10.1016/S0076-6879(09)64011-8 PMC4196316

[B12] Bayburt TH Sligar SG Membrane protein assembly into Nanodiscs FEBS Lett 2010 584 1721 1727 10.1016/j.febslet.2009.10.024 19836392 PMC4758813

[B13] Henderson R Unwin PN Three-dimensional model of purple membrane obtained by electron microscopy Nature 1975 257 28 32 10.1038/257028a0 1161000

[B14] Bayburt TH Grinkova YV Sligar SG Assembly of single bacteriorhodopsin trimers in bilayer nanodiscs Arch Biochem Biophys 2006 450 215 222 10.1016/j.abb.2006.03.013 16620766

[B15] Blanchette CD Cappuccio JA Kuhn EA Segelke BW Benner WH Chromy BA Coleman MA Bench G Hoeprich PD Sulchek TA Atomic force microscopy differentiates discrete size distributions between membrane protein containing and empty nanolipoprotein particles Biochim Biophys Acta 2008 1788 724 731 19109924 10.1016/j.bbamem.2008.11.019

[B16] Baas BJ Denisov IG Sligar SG Homotropic cooperativity of monomeric cytochrome P450 3A4 in a nanoscale native bilayer environment Arch Biochem Biophys 2004 430 218 228 10.1016/j.abb.2004.07.003 15369821

[B17] Das A Sligar SG Modulation of the cytochrome P450 reductase redox potential by the phospholipid bilayer Biochemistry 2009 48 12104 12112 10.1021/bi9011435 19908820 PMC2797566

[B18] Leitz AJ Bayburt TH Barnakov AN Springer BA Sligar SG Functional reconstitution of Beta2-adrenergic receptors utilizing self-assembling Nanodisc technology Biotechniques 2006 40 601 602 10.2144/000112169 16708760

[B19] Baker SE Hopkins RC Blanchette CD Walsworth VL Sumbad R Fischer NO Kuhn EA Coleman M Chromy BA Létant SE Hoeprich PD Adams MW Henderson PT Hydrogen production by a hyperthermophilic membrane-bound hydrogenase in water-soluble nanolipoprotein particles J Am Chem Soc 2009 131 7508 7509 10.1021/ja809251f 19449869

[B20] Driessen AJ Nouwen N Protein translocation across the bacterial cytoplasmic membrane Annu Rev Biochem 2008 77 643 67 10.1146/annurev.biochem.77.061606.160747 18078384

[B21] Alami M Dalal K Lelj-Garolla B Sligar SG Duong F Nanodiscs unravel the interaction between the SecYEG channel and its cytosolic partner SecA EMBO J 2007 26 1995 2004 10.1038/sj.emboj.7601661 17396152 PMC1852787

[B22] Katayama H Wang J Tama F Chollet L Gogol EP Collier RJ Fisher MT Three-dimensional structure of the anthrax toxin pore inserted into lipid nanodiscs and lipid vesicles Proc Natl Acad Sci USA 2010 107 3453 3457 10.1073/pnas.1000100107 20142512 PMC2840458

[B23] Raschle T Hiller S Yu TY Rice AJ Walz T Wagner G Structural and functional characterization of the integral membrane protein VDAC-1 in lipid bilayer nanodiscs J Am Chem Soc 2009 131 17777 17779 10.1021/ja907918r 19916553 PMC2793270

[B24] Bhattacharya P Grimme S Ganesh B Gopisetty A Sheng JR Martinez O Jayarama S Artinger M Meriggioli M Prabhakar BS Nanodisc-incorporated hemagglutinin provides protective immunity against influenza virus infection J Virol 2010 84 361 371 10.1128/JVI.01355-09 19828606 PMC2798435

[B25] Ryan RO Nanodisks: hydrophobic drug delivery vehicle Expert Opin Drug Deliv 2008 5 343 351 10.1517/17425247.5.3.343 18318655

[B26] Iovannisci DM Beckstead JA Ryan RO Targeting nanodisks via an apolipoprotein - single chain variable antibody chimera Biochem Biophys Res Commun 2009 379 466 469 10.1016/j.bbrc.2008.12.077 19114030 PMC2643875

[B27] Hartsel S Bolard J Amphotericin B: new life for an old drug Trends Pharmacol Sci 1996 17 445 449 10.1016/S0165-6147(96)01012-7 9014498

[B28] Oda MN Hargreaves P Beckstead JA Redmond KA van Antwerpen R Ryan RO Reconstituted high-density lipoprotein enriched with the polyene antibiotic, amphotericin B J Lipid Res 2006 47 260 267 10.1194/jlr.D500033-JLR200 16314670

[B29] Nguyen T-S Weers PMM Raussens V Wang Z Ren G Sulchek T Hoeprich PD Ryan RO Amphotericin B induces interdigitation of apolipoprotein stabilized nanodisk bilayers Biochim Biophys Acta 2008 1778 303 312 10.1016/j.bbamem.2007.10.005 17980702 PMC2254181

[B30] Nelson KG Bishop J Ryan RO Titus R Nanodisk-associated amphotericin B clears *Leishmania major *cutaneous infection in susceptible BALB/c mice Antimicrob Agents Chemother 2006 50 1238 1244 10.1128/AAC.50.4.1238-1244.2006 16569834 PMC1426947

[B31] Modolell M Choi BS Ryan RO Hancock M Titus RG Abebe T Hailu A Müller I Rogers ME Bangham CR Munder M Kropf P Local suppression of T cell responses by arginase-induced L-arginine depletion in nonhealing leishmaniasis PLoS Negl Trop Dis 2009 3 e480 10.1371/journal.pntd.0000480 19597544 PMC2703824

[B32] Soprano DR Qin P Soprano KJ Retinoic acid receptors and cancers Annu Rev Nutr 2004 24 201 221 10.1146/annurev.nutr.24.012003.132407 15189119

[B33] Altucci L Gronemeyer H The promise of retinoids to fight against cancer Nat Rev Cancer 2001 1 181 193 10.1038/35106036 11902573

[B34] Adamson PC All-Trans-Retinoic Acid Pharmacology and Its Impact on the Treatment of Acute Promyelocytic Leukemia Oncologist 1996 1 305 314 10388008

[B35] Guidoboni M Zancai P Cariati R Rizzo S Dal Col J Pavan A Gloghini A Spina M Cuneo A Pomponi F Bononi A Doglioni C Maestro R Carbone A Boiocchi M Dolcetti R Retinoic acid inhibits the proliferative response induced by CD40 activation and interleukin-4 in mantle cell lymphoma Cancer Res 2005 65 587 95 15695403

[B36] Kitareewan S Blumen S Sekula D Bissonnette RP Lamph WW Cui Q Gallagher R Dmitrovsky E G0S2 is an all-trans-retinoic acid target gene Int J Oncol 2008 33 397 404 18636162 PMC2597086

[B37] Altucci L Leibowitz MD Ogilvie KM de Lera AR Gronemeyer H RAR and RXR modulation in cancer and metabolic disease Nat Rev Drug Discov 2007 6 793 810 10.1038/nrd2397 17906642

[B38] Freemantle SJ Spinella MJ Dmitrovsky E Retinoids in cancer therapy and chemoprevention: promise meets resistance Oncogene 2003 22 7305 7315 10.1038/sj.onc.1206936 14576840

[B39] Takitani K Hino N Terada Y Kurosawa Y Koh M Inoue A Kawakami C Kuno T Tamai H Plasma all-trans retinoic acid level in neonates of mothers with acute promyelocytic leukemia Acta Haematol 2005 114 167 169 10.1159/000087893 16227682

[B40] Redmond KA Nguyen T-S Ryan RO All-trans retinoic acid nanodisks Int J Pharm 2007 339 246 250 10.1016/j.ijpharm.2007.02.033 17412536 PMC2045639

[B41] Singh AT Evens AM Anderson RJ Beckstead JA Sankar N Sassano A Bhalla S Yang S Platanias LC Forte TM Ryan RO Gordon LI All trans retinoic acid nanodisks enhance retinoic acid receptor mediated apoptosis and cell cycle arrest in mantle cell lymphoma Br J Haematol 2010 150 158 169 20507312 10.1111/j.1365-2141.2010.08209.x PMC2907750

[B42] Bertoni F Ponzoni M The cellular origin of mantle cell lymphoma Int J Biochem Cell Biol 2007 39 1747 1753 10.1016/j.biocel.2007.04.026 17574898

[B43] Epstein J Sanderson IR Macdonald TTX Curcumin as a therapeutic agent: the evidence from in vitro, animal and human studies Br J Nutr 1994 26 1 13 10.1017/S0007114509993667 20100380

[B44] Hatcher H Planalp R Cho J Torti FM Torti SV Curcumin: from ancient medicine to current clinical trials Cell Mol Life Sci 2008 65 1631 1652 10.1007/s00018-008-7452-4 18324353 PMC4686230

[B45] Jurenka JS Anti-inflammatory properties of curcumin, a major constituent of *Curcuma longa*: a review of preclinical and clinical research Alternative Medicine Review 2009 14 141 153 19594223

[B46] Ghosh M Singh ATK Xu W Sulchek T Gordon LI Ryan RO Curcumin nanodisks: formulation and characterization Nanomedicine 2010 in press 20817125 10.1016/j.nano.2010.08.002 PMC3030927

[B47] De Leon-Rodriguez LM Lubag AJ Malloy CR Martinez GV Gillies RJ Sherry AD Responsive MRI agents for sensing metabolism in vivo Acc Chem Res 2009 42 948 957 10.1021/ar800237f 19265438 PMC2713815

[B48] Skajaa T Cormode DP Falk E Mulder WJ Fisher EA Fayad ZA High-density lipoprotein-based contrast agents for multimodal imaging of atherosclerosis Arterioscler Thromb Vasc Biol 2010 30 169 176 10.1161/ATVBAHA.108.179275 19815819 PMC2826843

[B49] Frias JC Ma Y Williams KJ Fayad ZA Fisher EA Properties of a versatile nanoparticle platform contrast agent to image and characterize atherosclerotic plaques by magnetic resonance imaging Nano Lett 2006 6 2220 2224 10.1021/nl061498r 17034087

[B50] Cormode DP Briley-Saebo KC Mulder WJ Aguinaldo JG Barazza A Ma Y Fisher EA Fayad ZA An ApoA-I mimetic peptide high-density-lipoprotein-based MRI contrast agent for atherosclerotic plaque composition detection Small 2008 4 1437 1444 10.1002/smll.200701285 18712752

[B51] Cormode DP Skajaa T van Schooneveld MM Koole R Jarzyna P Lobatto ME Calcagno C Barazza A Gordon RE Zanzonico P Fisher EA Fayad ZA Mulder WJ Nanocrystal core high-density lipoproteins: a multimodality contrast agent platform Nano Lett 2008 8 3715 3723 10.1021/nl801958b 18939808 PMC2629801

[B52] Chen W Vucic E Leupold E Mulder WJ Cormode DP Briley-Saebo KC Barazza A Fisher EA Dathe M Fayad ZA Incorporation of an apoE-derived lipopeptide in high-density lipoprotein MRI contrast agents for enhanced imaging of macrophages in atherosclerosis Contrast Media Mol Imaging 2008 3 233 242 10.1002/cmmi.257 19072768

[B53] Chen W Jarzyna PA van Tilborg GA Nguyen VA Cormode DP Klink A Griffioen AW Randolph GJ Fisher EA Mulder WJ Fayad ZA RGD peptide functionalized and reconstituted high-density lipoprotein nanoparticles as a versatile and multimodal tumor targeting molecular imaging probe FASEB J 2010 24 1689 1699 10.1096/fj.09-139865 20075195 PMC2874482

[B54] Fischer NO Blanchette CD Chromy BA Kuhn EA Segelke BW Corzett M Bench G Mason PW Hoeprich PD Immobilization of his-tagged proteins on nickel-chelating nanolipoprotein particles Bioconjug Chem 2009 20 460 465 10.1021/bc8003155 19239247

[B55] Fischer NO Infante E Ishikawa T Blanchette CD Bourne N Hoeprich PD Mason PW Conjugation to nickel-chelating nanolipoprotein particles increases the potency and efficacy of subunit vaccines to prevent West Nile encephalitis Bioconjug Chem 2010 21 1018 1022 10.1021/bc100083d 20509624 PMC2918428

[B56] Borch J Torta F Sligar SG Roepstorff P Nanodiscs for immobilization of lipid bilayers and membrane receptors: kinetic analysis of cholera toxin binding to a glycolipid receptor Anal Chem 2008 80 6245 6252 10.1021/ac8000644 18616345

[B57] Matsuo Y Miura S Kawamura A Uehara Y Rye KA Saku K Newly developed reconstituted high-density lipoprotein containing sphingosine-1-phosphate induces endothelial tube formation Atherosclerosis 2007 194 159 168 10.1016/j.atherosclerosis.2006.10.020 17118370

[B58] Thaxton CS Daniel WL Giljohann DA Thomas AD Mirkin CA Templated spherical high density lipoprotein nanoparticles J Am Chem Soc 2009 131 1384 1385 10.1021/ja808856z 19133723 PMC2843502

[B59] Peters-Libeu CA Newhouse Y Hall SC Witkowska HE Weisgraber KH Apolipoprotein E-dipalmitoylphosphatidylcholine particles are ellipsoidal in solution J Lipid Res 2007 48 1035 1044 10.1194/jlr.M600545-JLR200 17308333

[B60] Wu Z Gogonea V Lee X Wagner MA Li XM Huang Y Undurti A May RP Haertlein M Moulin M Gutsche I Zaccai G Didonato JA Hazen SL Double superhelix model of high density lipoprotein J Biol Chem 2009 284 36605 36619 10.1074/jbc.M109.039537 19812036 PMC2794775

[B61] Skar-Gislinge N Simonsen JB Mortensen K Feidenhans'l R Sligar SG Lindberg B Møller Bjørnholm T Arleth L Elliptical structure of phospholipid bilayer nanodiscs encapsulated by scaffold proteins: casting the roles of the lipids and the protein J Am Chem Soc 2010 132 13713 13722 10.1021/ja1030613 20828154 PMC4120756

